# Chronic post-stroke aphasia severity is determined by fragmentation of residual white matter networks

**DOI:** 10.1038/s41598-017-07607-9

**Published:** 2017-08-15

**Authors:** Barbara K. Marebwa, Julius Fridriksson, Grigori Yourganov, Lynda Feenaughty, Chris Rorden, Leonardo Bonilha

**Affiliations:** 1 Department of Neurology, Medical University of South Carolina, Charleston, SC USA; 2 Department of Communication Sciences and Disorders, University of South Carolina, Columbia, SC USA; 3 Department of Psychology, University of South Carolina, Columbia, SC USA; 4 School of Communication Sciences and Disorders, The University of Memphis, Memphis, Tennessee USA

## Abstract

Many stroke survivors with aphasia in the acute period experience spontaneous recovery within the first six months after the stroke. However, approximately 30–40% sustain permanent aphasia and the factors determining incomplete recovery are unclear. Suboptimal recovery may be influenced by disruption of areas seemingly spared by the stroke due to loss of white matter connectivity and network integrity. We reconstructed individual anatomical whole-brain connectomes from 90 left hemisphere stroke survivors using diffusion MR images. We measured the modularity of the residual white matter network organization, the probability of brain regions clustering together, and the degree of fragmentation of left hemisphere networks. Greater post-stroke left hemisphere network fragmentation and higher modularity index were associated with more severe chronic aphasia, controlling for the size of the stroke lesion. Even when the left hemisphere was relatively spared, subjects with disorganized community structure had significantly worse aphasia, particularly when key temporal lobe regions were isolated into segregated modules. These results suggest that white matter integrity and disorganization of neuronal networks could be important determinants of chronic aphasia severity. Connectome white matter organization measured through modularity and other topological features could be used as a personalized variable for clinical staging and aphasia treatment planning.

## Introduction

Human communication relies on complex interactions of higher-order processes, such as general knowledge, memory, semantic association, syntax, and phonological processing. Taken together, key cortical regions need not only to be preserved, but also connected and integrated into a neural network in order to permit language processing.

Stroke is the leading cause of long term language impairments (aphasia) in adults^1^. However, many stroke survivors with aphasia in the acute phase experience spontaneous recovery within the first six months after the stroke. Nonetheless, approximately 30–40% do not recover fully and experience aphasia for the rest of their lives^2^. Even though ischemic stroke may lead to necrotic damage affecting specific brain areas, the functional impairment after stroke can be exacerbated by dysfunction of seemingly spared areas^3^.

The neurobiological bases for loss of function in remote and spared areas are not completely understood. However, extensive work on disconnection syndromes, including from our group^4–6^, have demonstrated that white matter loss and cortical disconnection can extend beyond the stroke lesion^3^. Importantly, the degree of white matter disconnection of Broca’s area is an independent predictor of naming impairments after a stroke, controlling for the degree of cortical ischemic damage^7, 8^. Furthermore, residual anatomical connectivity of spared areas plays a significant role in therapy-related improvement in object naming in subjects with aphasia^9^.

Nonetheless, it is still unclear whether post-stroke white matter damage can be used as personalized predictor of chronic aphasia severity.

Our group has recently described how the comprehensive map of white matter connectivity (the connectome) can be measured in stroke survivors by combining innovations in image registration, probabilistic tractography, diffusion tensor imaging and statistical assessment of residual networks^10, 11^. While understanding the effect of single elements such as node strength or degree, can explain some of the behavioral impairments after stroke, further understanding the complex topological organization of these elements may provide a valuable panoramic perspective that may not be captured otherwise.

Examining the community organization via modularity is one such approach to assess the mesoscale organization in the network. Therefore, in this study, we applied connectome methods to test the hypothesis that post-stroke residual white matter connectivity in chronic stroke survivors is associated with long-lasting aphasia severity. We employed graph theory methods to assess the community structure of white matter networks and we hypothesized that the fragmentation of the connectivity structure of the networks in the dominant hemisphere, even when cortical regions are relatively spared, would be associated with more severe aphasia.

## Methods

### Participants

We recruited 90 participants, (mean age 58.8 ± 12.1 years, 34 women) with a single left hemisphere ischemic or hemorrhagic stroke at least 6 months before the study (mean 42.8 ± 50 months post stroke). Participants were included in the study if they did not have a history of any other neurological illness apart from the stroke, could follow simple instructions, and were MRI compatible. All participants were right handed. The cohort of participants included in this study was also reported in a previous study by our group^11^. The study was approved by the Institutional Review Boards at the Medical University of South Carolina and at the University of South Carolina. Written informed consent was obtained from all participants or their legal guardians, as approved by our institutions’ IRB. All methods were performed in accordance with guidelines and regulations from our institutions’ IRB.

### Behavioral Evaluation

All the participants underwent language assessment using the Western Aphasia Battery (WAB-R)^12^. The variable of interest to the current study was the WAB Aphasia Quotient (WAB-AQ), which yields a global measure of aphasia severity on a scale of 0–100, with lower scores indicating worse aphasia. WAB-AQ reflects overall severity of language impairment in aphasia and is derived from various subtest scores including spontaneous speech fluency, auditory comprehension, speech repetition, and naming. Each subtest score was obtained by combining the data from its corresponding categories and calculated in accordance with the WAB-R manual. Aphasia types were classified according to the WAB. The following aphasia types were observed in our participant sample: anomic (26 participants); Broca’s (30 participants); conduction (9 participants); global (8 participants); Wernicke’s (5 participants); no aphasia (12 participants) with WAB-AQ > 93.7; a cut-off typically applied in clinical grounds, but may otherwise exclude individuals with milder deficits.

### Image Acquisition

MRI scanning was performed within two days of behavioral testing. Images were acquired on a Siemens Trio 3 T scanner equipped with a 12-element head coil located at the University of South Carolina. In this study, we used whole brain T1-weighted, T2-weighted and Diffusion EPI images collected from each patient.

1. T1-weighted image utilizing an MP-RAGE sequence with 1 mm isotropic voxels, a 256 × 256 matrix size, and a 9-degree flip angle. For the first 25 individuals we used a 160 slice sequence with TR = 2250 ms, TI = 900 ms, TE = 4.52 ms. For the latter 65 individuals we used a 192 slice sequence with TR = 2250 ms, TI = 925 ms, TE = 4.15 with parallel imaging (GRAPPA = 2, 80 reference lines). Each of these scans required approximately 7 minutes to acquire.
2. T2-weighted image using a sampling perfection with application optimized contrasts using a different flip angle evolution (3D-SPACE) sequence. This 1 mm isotropic 3D TSE scan uses a TR = 2800 ms, a TE of 402 ms, variable flip angle, 256 × 256 matrix scan with 192 slices, using parallel imaging (GRAPPA × 2, 120 reference lines).
3. Diffusion EPI scan which varied in terms of b-value (s/mm^2^), spatial resolution and other parameters across participants. Fifty-two participants had a sequence with 65 isotropic (2.0 mm) volumes (x1 B = 0, × 64 B = 1000), TR = 7700 ms, TE = 90 ms, 112 × 112 matrix, with parallel imaging GRAPPA = 2, 60 contiguous slices. Thirty-eight individuals had a monopolar sequence with 82 isotropic (2.3 mm) volumes (x10 B = 0, × 72 B = 1000), TR = 4987 ms, TE = 79.2 ms, 90 × 90 matrix, with parallel imaging GRAPPA = 2, 44 contiguous slices. This sequence was acquired in two series (41 volumes in each series) with opposite phase encoding allowing us to spatially undistort the images with TOPUP^13^.

### Lesion Mapping

Lesions were manually drawn on each individuals T2 scan by a neurologist (LB). The T2 scan was co-registered with the individual’s T1 scan with the transforms applied to the lesion map. The T1 scans were warped to standard space using enantiomorphic segmentation-normalization^14^, with these transforms applied to the lesion maps. These normalized lesion maps were used to compute the proportion injury to each of the 189 regions in the JHU as a ratio of lesioned voxels to the total number of voxels in that region.

### Brain parcellation

Normalized brains were segmented into 189 regions using the Johns Hopkins University (JHU) brain atlas^15^. We aligned the anatomical brain atlas containing the JHU parcellation with each individual’s T1-weighted images. The T1-weighted images were segmented into probabilistic grey and white matter maps, and the grey matter map was divided into regions according to the atlas. We then computed the percentage of damage for each grey matter region as a ratio of lesioned voxels to the total number of voxels in that region.

### Anatomical connectome construction

Each participant’s individual connectome was built using the following steps: 1) T1 weighted images were segmented into probabilistic grey and white matter maps using SPM12’s unified segmentation-normalization, 2) the probabilistic grey matter map was divided into the JHU anatomical regions using the parcellation scheme described above, 3) the white matter and grey matter parcellation maps were registered into the diffusion imaging (DTI) space, 4) pairwise probabilistic DTI fiber tracking was computed for grey matter regions, 5) the weight of each pairwise connectivity link was determined based on the number of streamlines connecting the grey matter region pair, corrected by distance travelled by each streamline and by the total volume of the connected regions, and 6) a weighted adjacency matrix **M** of size 189 × 189 was constructed for each participant, with **M**_**i**,**j**_ representing the weighted link between ROI **i** and ROI **j**.

The T2-weighted image (co-registered into the T1-weighted image) was normalized into the B0 non-diffusion image (from the diffusion MRI sequence); this spatial transform was applied to register the probabilistic white and grey matter maps (the latter divided into JHU regions of interest) as well as the stroke lesion into the diffusion MRI space, where all subsequent calculations were performed.

Tractography was estimated through FDT’s probabilistic method^16^ with FDT’s BEDPOST being used to assess default distributions of diffusion parameters at each voxel, and probabilistic tractography was performed using FDT’s probtrackX (parameters: 5000 individual pathways drawn through the probability distributions on principle fiber direction, curvature threshold set at 0.2, 200 maximum steps, step length 0.5 mm, and distance correction). The waypoint mask was set as the white-matter probabilistic map excluding the stroke lesion, ensuring that the subsequent weighted connectivity matrix is composed of only the surviving connections. The weighted connectivity between the regions *i* and *j* was defined as the number of probabilistic streamlines arriving at region *j* when *i* was seeded, averaged with the number of probabilistic streamlines arriving at region *i* when *j* was seeded. The connection weight was corrected based on the distance travelled by the streamlines connecting *i* and *j* (probtrackX’s “distance correction”). The number of streamlines connecting each pair of regions was further divided by the sum of the volumes of these regions, giving the number of connections per unit surface. The distance correction is essential to eliminate linear bias towards longer fibers, and the volume correction avoids oversampling of larger ROIs compared to ROIs with smaller areas^17^. We did not perform a network density correction because as previously demonstrated by our group^18^, in a weighted or non-binarized network, network density does not affect network properties as all possible connections are taken into account and scaled based on their weight.

Each individual connectome was represented by a 189 × 189 matrix, where the nodes corresponded to the JHU anatomical regions, and the edges corresponded to the anatomical connectivity between the nodes. For this study, our analyses were restricted to 57 × 57 matrices that included only grey matter regions (i.e., ventricles and white matter regions were excluded).

Succinctly, the following procedures were performed: the lesion was manually drawn on a T2 weighted image by a rater who was blinded to the WAB-AQ, the T2 and the T1 weighted image were co-registered and the T1 weighted image was spatially registered (non-linearly normalized) into standard space using an enantiomorphic segmentation-normalization procedure. Then the transformation matrix (T1 to standard space) was used to transform the JHU atlas into native T1 space, and a non-linear normalization procedure was used to register the T1 to the B0 image, and this transformation matrix was used to transform the JHU atlas from T1 to diffusion space. The same procedure was used to transform the lesion (in T1 space) to diffusion space, and finally fiber tracking was performed using the JHU ROIs while excluding the lesion mask.

### Community Detection

Each connectome created above was partitioned into communities or modules by optimizing Newman’s modularity algorithm^19^, implemented in the Brain Connectivity Toolbox^20^ (e.g. [Ci,Q] = modularity_und(W), where W is the weighted undirected connectivity matrix; gamma was maintained at the default gamma = 1). Modularity (Q) is a value that quantifies the strength of the network’s modular organization by identifying groups of nodes that have a stronger intra-community coherence than inter-community coherence, and is defined as

1 $$Q=\sum _{i=1}^{m}({e}_{ii}-{a}_{i}^{2})$$

where **m** is the total number of modules, ***e***_***ii***_ is the fraction of edges in the network that connect nodes that occur within the same module ***i***, and ***a***_***i***_ is the fraction of edges connecting a node in module **i** to any other random node, such that if the modules were assigned randomly, then **e**_**ij**_ **= a**_**i**_ **a**_**j**_. Modularity values are positive if the number of edges within modules exceeds the number of edges expected by a chance distribution of edges between nodes regardless of modules^19^.

### Statistical analysis

For each subject, in the left and right hemisphere, we extracted the modularity score, and the optimal community structure, which indicates to which communities each ROI belongs. Since two different sequences were used to acquire the DTI data, we first ran an unpaired two-tailed t-test and determined there was no significant difference in the modularity scores acquired from the two groups, left hemisphere (p = 0.3340), right hemisphere (p = 0.1455). Due to stochasticity of network partitioning which may lead to assignment of ROIs to different communities with every run, we performed 100 optimizations of the modularity quality function for each connectivity matrix and created a community affiliation matrix **A**, from the optimal community structure vector. **A**_**ij**_ represented the probability that region **A**_**i**_ and **A**_**j**_ are consistently grouped in the same community over 100 iterations. We then calculated the mean of all entries in the upper triangular community affiliation matrix, to obtain the left and right hemisphere community affiliation index (C).

For each subject, we quantified how intact the community structure of the left hemisphere was, compared to the right hemisphere via a ratio of the right to left community affiliation index, which we called the *fragmentation index* (FI) defined as

2 $$FI=\frac{R{H}_{C}-\,L{H}_{C}}{R{H}_{C}+\,L{H}_{C}\,}$$

where RH_C_ is the right hemisphere community affiliation index, and LH_C_ is the left hemisphere community affiliation index.

We then performed a one-tailed Pearson correlation analysis, since we had an a priori expectation of effect in one direction - higher modularity to correspond to lower behavioral scores, to evaluate relationship between modularity, community affiliation index, and fragmentation index with WAB-AQ scores. We also calculated partial correlations controlling for whole brain grey and white matter damage, and for damage in a subnetwork of language specific regions as defined by Fedorenko and colleagues^21^. We further calculated the sum of weighted links to each node; the node strength. The mean node strength of left hemisphere nodes was then correlated with WAB-AQ.

To determine which pairs of nodes should be in the same module for better WAB-AQ score, we calculated for every entry in the left hemisphere community affiliation matrix **A**_**ij**_, an unpaired one-tailed t-test that compared the WAB-AQ scores for participants that had a community affiliation index of 1 against those that had an index < 1. The t-test was run only if both groups had at least 5 participants. To control for damage, the analysis was further restricted to pairs of regions that were at least 50% preserved for each individual. The p-values were Bonferroni corrected at p ≤ 0.05.

In order to assess the relationship between modularity and regional grey matter hubs, the number of preserved hubs in the left hemisphere was assessed. Network hubs were defined in accordance with rich club networks^22^ as previously applied to lesion brains and aphasia by our group^23^, by identifying nodes with high degree that were also more densely connected to other high degree nodes than would be expected by chance.

We performed multiple linear regression analyses to evaluate the relationship between WAB-AQ and cortical damage, modularity, and the number of hubs. In the first model, WAB-AQ was defined as the dependent variable, with damage as the independent variable. The second model had WAB-AQ as the dependent variable, and damage and number of hubs as independent variables. The third model had WAB-AQ as the dependent variable, and damage and modularity as independent variables. Adjusted R^2^ indicated the explanatory power of the model with statistical significance set at p < 0.05.

### Contributions of the domain general and language specific networks

To determine which community structures were associated with aphasia severity, we performed subsequent analyses evaluating a sub-network composed of 9 language specific and 8 domain general regions of the networks as defined by Fedorenko and colleagues^21^. Language specific regions included the posterior segment of the middle frontal gyrus (MFG), the inferior frontal gyrus opercularis, inferior frontal gyrus triangularis, angular gyrus (AG), superior temporal gyrus (STG), pole of the superior temporal gyrus, middle temporal gyrus (MTG), posterior superior temporal gyrus (PSTG), and the posterior middle temporal gyrus (PMTG) which corresponds to PSMG in the JHU atlas. We also included the posterior inferior temporal gyrus (PITG) which corresponds to PSIG in the JHU atlas. Domain general regions included the posterior segment of the superior frontal gyrus (SFG), the dorsal prefrontal cortex of the middle frontal gyrus (MFG-DPFC), inferior frontal gyrus orbitalis, precentral gyrus (PrCG), superior parietal gyrus (SPG), supramarginal gyrus (SMG), posterior cingulate gyrus (PCC) and the insular. For every node in the language specific and domain general networks we calculated the frequency with which the node occurred in the same module with other regions in the brain for each subject, in other words, when they were not fragmented. We then correlated this value with WAB-AQ scores to determine the correlation between the composition of the modules and aphasia severity.

In order to assess the strength of the connections within the communities, we further selected the traditional language regions: Broca’s area (inferior frontal gyrus opercularis and inferior frontal gyrus triangularis), and Wernicke’s area (superior temporal gyrus), and for each subject, extracted modules that contained these regions. We then calculated the average intra-modular degree for the modules containing these regions, and the participation coefficient for the three ROIs, which were then correlated with WAB-AQ, controlling for grey matter damage to the ROIs contained in each module.

Statistical analyses were performed in MATLAB Release 2015b.

## Results

### Relationship between Aphasia Severity and Modularity (Q)

**Figure 1:**
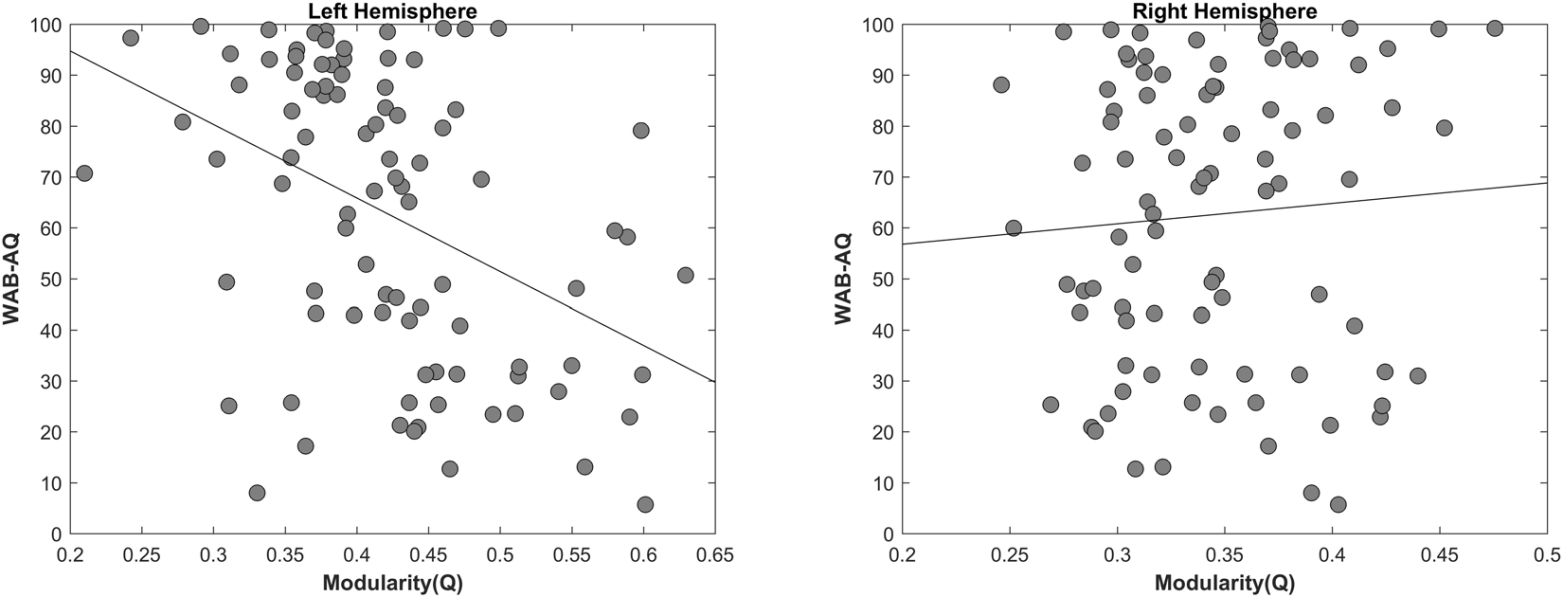
Correlation between modularity and aphasia severity in the left hemisphere (r = −0.4215, p < 0.00001), right hemisphere (r = 0.0698, p = 0.5135).

**Figure 2:**
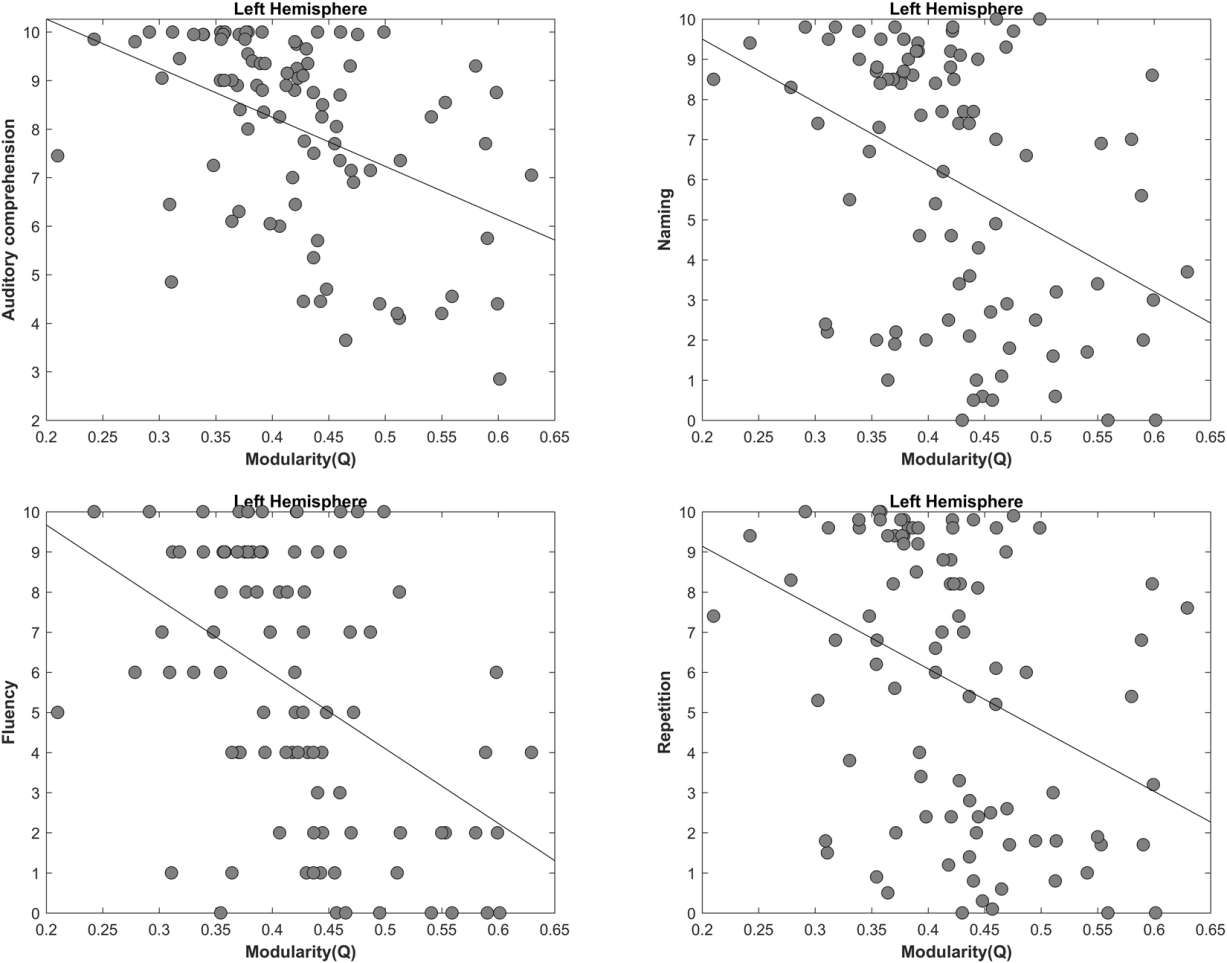
Correlation between left hemisphere modularity and subsets of WAB-AQ: Auditory comprehension (r = −0.4345, p < 0.00001), Fluency (r = −0.4561, p < 0.00001), Naming (r = −0.3948, p < 0.00001), and Repetition (r = −0.3644, p < 0.00001).

The mean aphasia quotient (WAB-AQ) was 62.6 ± 28.9. As indicated in the left panel of Fig. 1, left hemisphere modularity (Q) was significantly correlated with WAB-AQ scores such that greater modularity was associated with worse aphasia severity (r = −0.42, p < 0.00001) - partial correlation controlling for ROI-specific proportion of damage in the left hemisphere (r = −0.21, p = 0.0246), partial correlation controlling for language specific network damage in the left hemisphere (r = −0.21, p = 0.0225), partial correlation controlling for white matter damage in the left hemisphere (r = −0.28, p = 0.0044). Right hemisphere modularity (Q) was not significantly correlated with WAB-AQ (see right panel of Fig. 1). Figure 2 shows the correlation between left hemisphere modularity and WAB subscores: auditory comprehension, fluency, object naming, and repetition. There was no significant correlation between the whole brain modularity score and WAB-AQ (r = 0.1445, p = 0.1742), or any of the WAB subscores.

### Relationship between Aphasia Severity and mean node strength

The left hemisphere mean node strength was significantly correlated to WAB-AQ (r = 0.3625, p < 0.0001), but did not survive partial correlation controlling for the grey and white matter damage. Supplementary Figures 3 and 4 shows the relationship between WAB-AQ (and WAB subscores) and node strength.

### Relationship between Aphasia severity and Community Affiliation Index (C)

**Figure 3:**
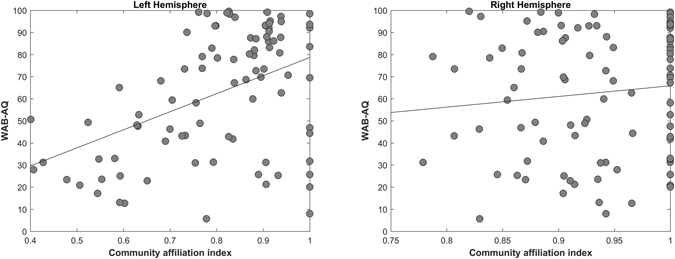
Correlation between community affiliation index (C) and aphasia severity. Left Hemisphere (r = 0.4438, p < 0.00001), Right Hemisphere (r = 0.12, p = 0.3144).

The left hemisphere community affiliation index was significantly correlated with WAB-AQ (r = 0.44, p < 0.00001), Fig. 3 left panel). The direction of the effect indicates that subjects whose connectomes exhibited more consistent left-hemispheric node-community assignments across optimizations of the clustering algorithm had higher WAB-AQ.

Aphasia severity and community affiliation index was not significantly correlated when controlling grey or white matter damage. There right hemisphere community affiliation index was not significantly correlated with WAB-AQ (Fig. 3 right panel).

### Relationship between Aphasia severity and Fragmentation Index (FI)

**Figure 4:**
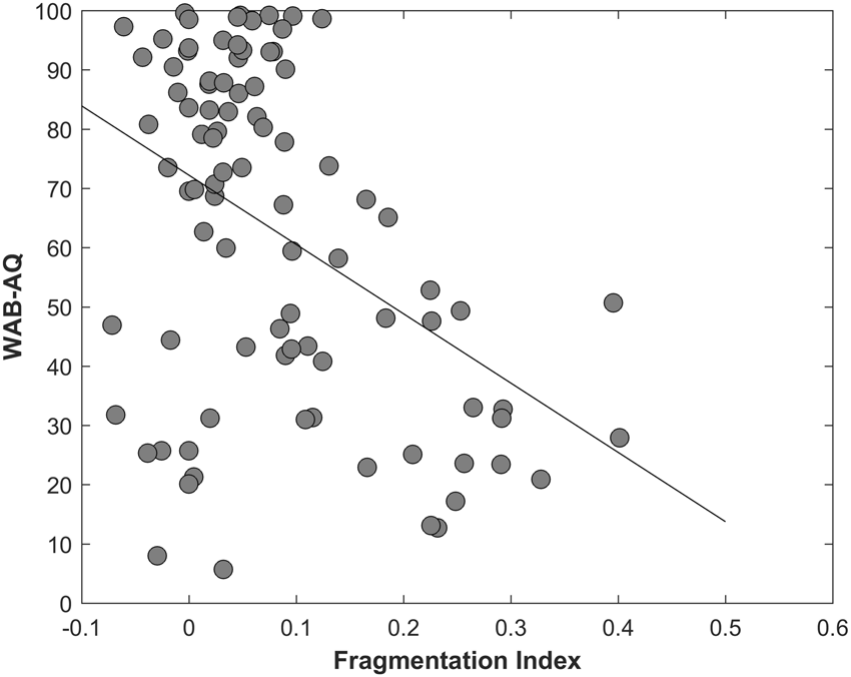
Correlation between Fragmentation index and aphasia severity (r = −0.4302, p < 0.0001).

There was a significant negative correlation between the fragmentation index and WAB-AQ (r = −0.43, p < 0.0001, Fig. 4), indicating that subjects with more fragmented left hemispheres had more severe aphasia.

**Figure 5:**
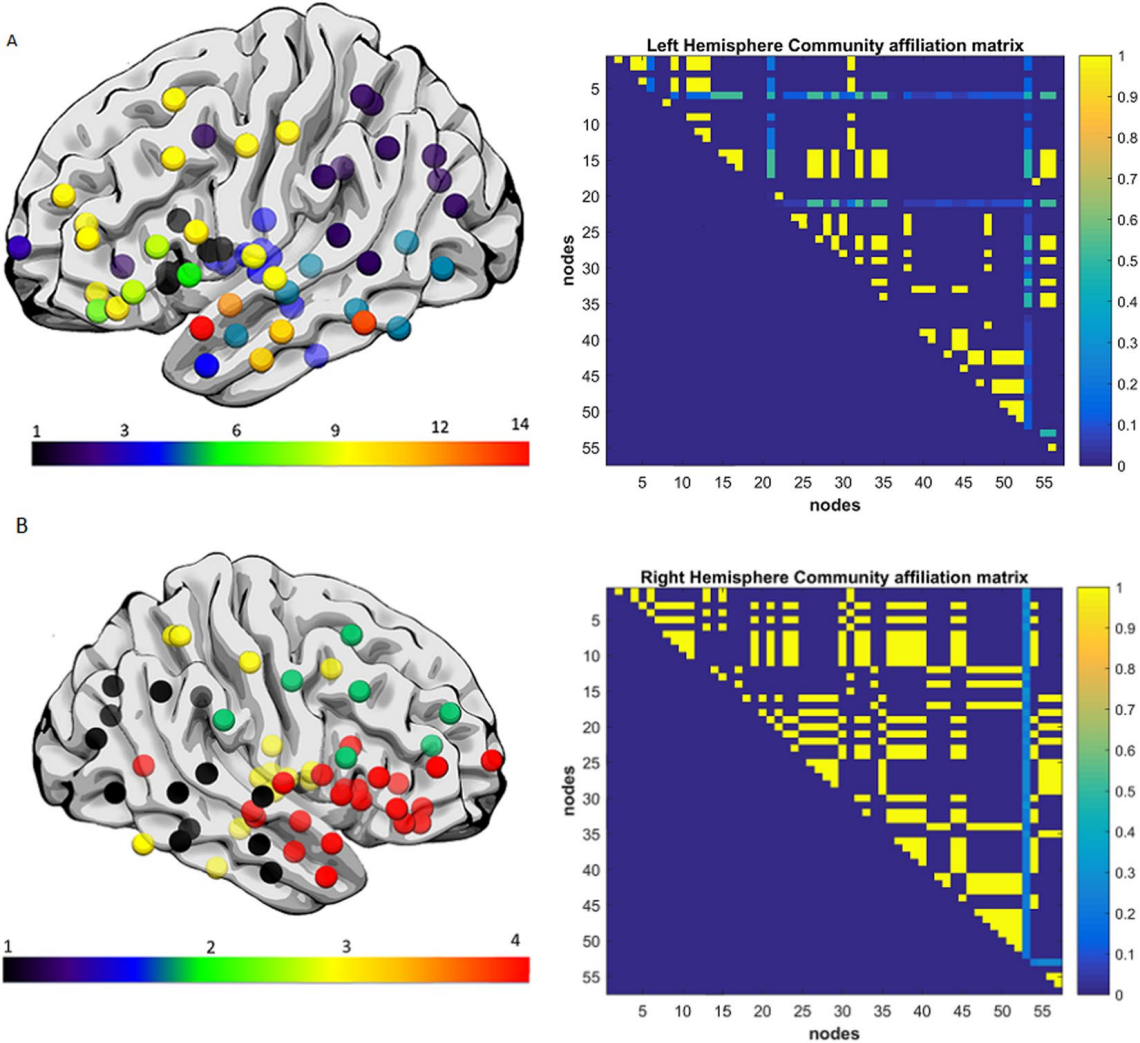
(**A**) Exemplar data Subject 1, left hemisphere lateral view, each color represents a single community. Note the fragmentation of the fronto-parietal and middle-temporal networks with relative disconnection between the frontal and subcortical regions, which are grouped into different modules. Subject 1 had a lesion volume of 359.4 cm^3^, percent white matter damage of 0.168, and a WAB-AQ score of 48.1. (Supplementary Table S2 shows the labels associated with the nodes on the community affiliation matrix). Note the fragmentation, unstable clustering shown and missing nodes in the community affiliation matrix compared to the right hemisphere matrix. (**B**) Exemplar data Subject 1, right hemisphere lateral view, each color represents a single community. There was no apparent fragmentation, and the network was divided into 4 communities. The community affiliation matrix also showed stable clusters over 100 runs.

**Figure 6:**
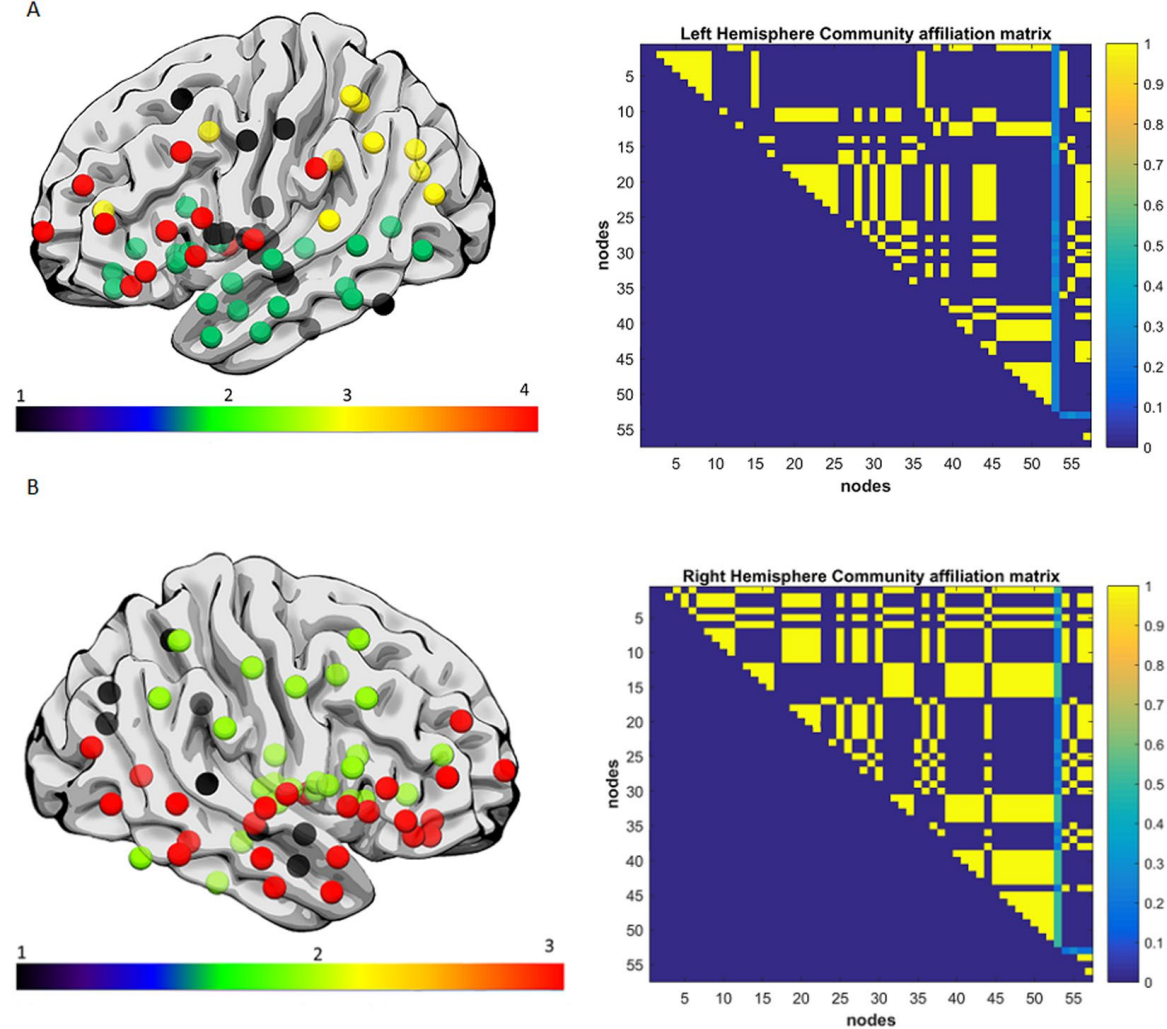
(**A**) Exemplar data Subject 2, each color represents a single community left hemisphere lateral view. Both hemispheres did not reveal dramatic fragmentation patterns. Subject 2 had a lesion volume of 76.1 cm^3^, percent white matter damage of 0.099, and a WAB-AQ score of 88.1. The community affiliation matrix showed relatively stable clustering over 100 runs. (**B**) Exemplar data Subject 2, right hemisphere lateral view, each color represents a single community, and again there was no apparent fragmentation, and the network was divided into 4 communities. The community affiliation matrix showed stable clustering over 100 runs.

**Figure 7:**
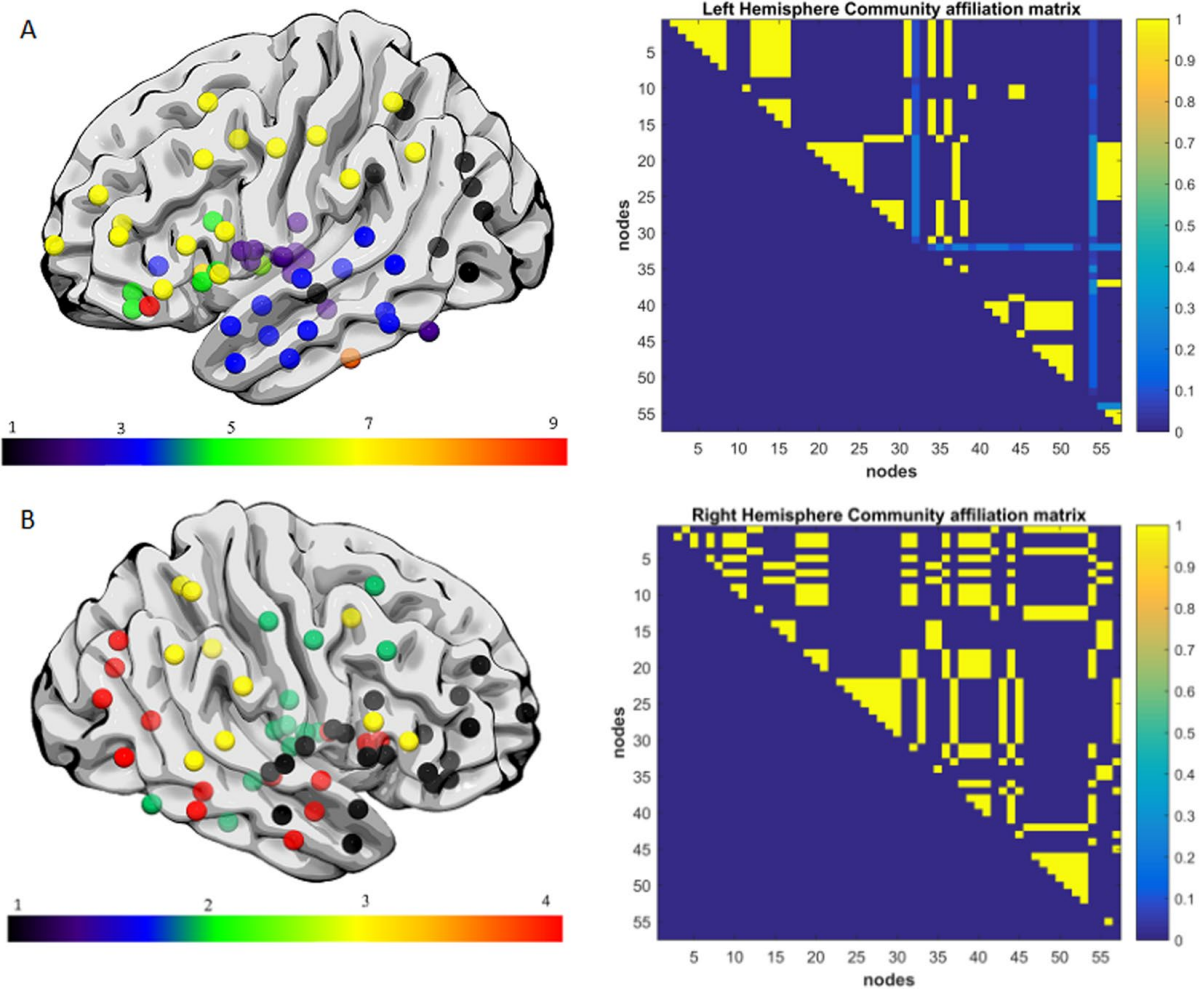
(**A**) Exemplar data Subject 3, each color represents a single community left hemisphere lateral view. There was marked fragmentation of the inferior frontal and middle-temporal networks with relative disconnection between the frontal and subcortical regions, with the left hemisphere grouping into 9 modules. Subject 3 had a lesion volume of 99.24 cm^3^, percent white matter damage of 0.096, and a WAB-AQ score of 58.2. There was unstable clustering and missing nodes in the community affiliation matrix compared to the right hemisphere matrix. (**B**) Exemplar data Subject 3, right hemisphere lateral view, each color represents a single community. There was no apparent fragmentation, and the network was divided into 4 communities. The community affiliation matrix showed relatively stable clustering over 100 runs.

**Figure 8:**
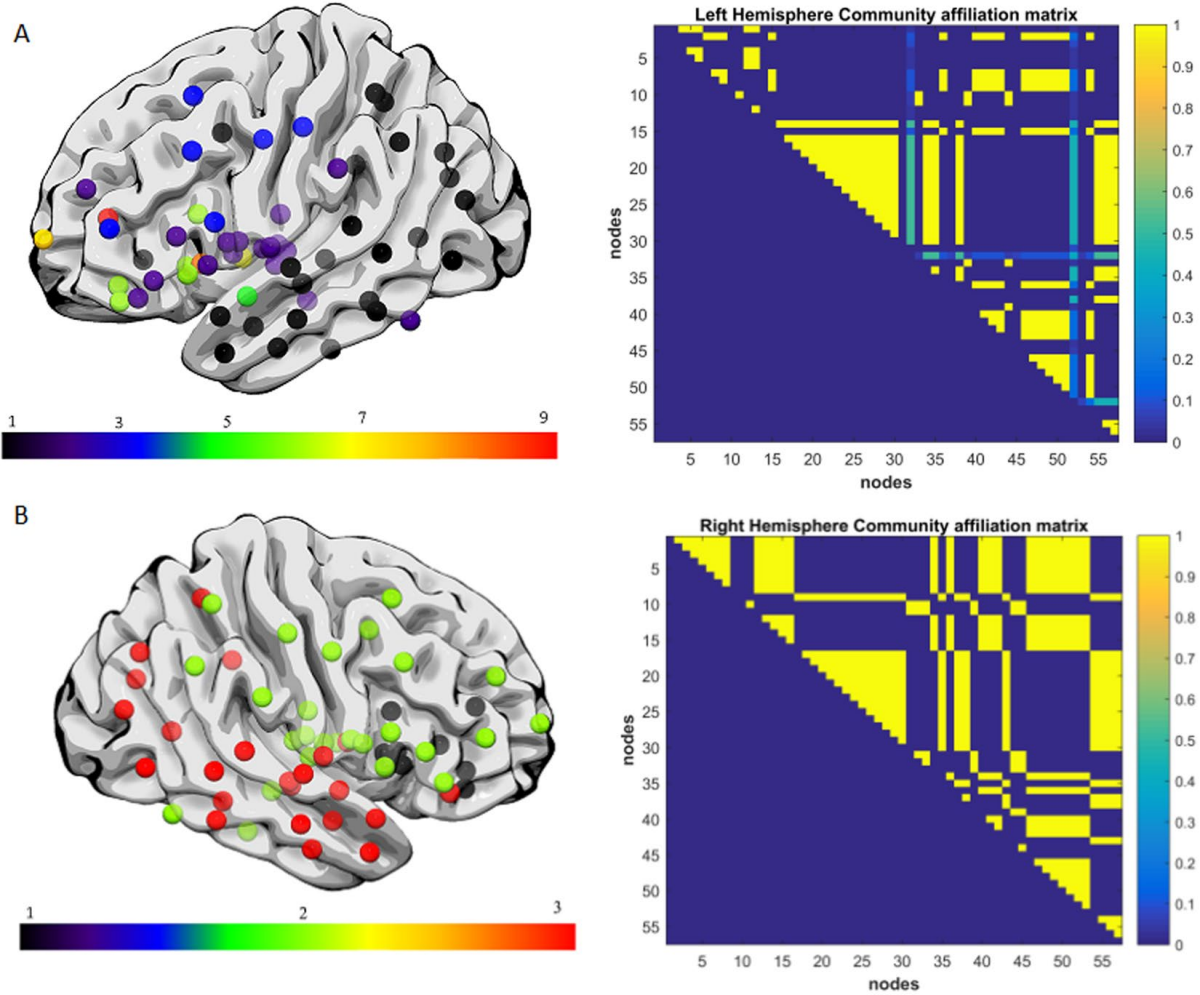
(**A**) Exemplar data Subject 4, each color represents a single community left hemisphere lateral view. Note the fragmentation of the fronto-parietal, inferior frontal and middle-temporal networks with the hemisphere grouping into 9 modules. Subject 4 had a lesion volume of 206.36 cm^3^, percent white matter damage of 0.035, and a WAB-AQ score of 41.8. Note the missing nodes and unstable clustering in the community affiliation matrix. (**B**) Exemplar data Subject 4, right hemisphere lateral view, each color represents a single community. There was no apparent fragmentation, and the network was divided into 3 communities. The community affiliation matrix also showed stable clustering over 100 runs.

Aphasia severity and fragmentation index was not significantly correlated when controlling grey matter damage, but was significantly correlated when controlling for white matter damage (r = −0.22, p = 0.0175). To illustrate the network fragmentation, Figs 5 and 6 show two example participants with very different lesion volumes (subject 1: 359.4 cm^3^ percent white matter damage: 0.168; subject 2: 76.1 cm^3^, percent white matter damage: 0.099). The impact of the lesion into the white matter fragmentation is remarkable. Subject 1’s left hemisphere was partitioned into 14 modules. The fronto-parietal and middle-temporal networks are highly fragmented, with relative disconnection between the frontal and subcortical regions, which are grouped into different modules (Fig. 5A). The right panel of Fig. 5A. shows the left hemisphere community affiliation matrix of subject 1, which is visibly sparser compared to the right hemisphere, indicating an unstable clustering with very few nodes consistently grouped in the same modules over 100 runs. Figure 5B is the same subject’s right hemisphere, which was partitioned into 4 groups, and displays significantly less fragmentation, and a more stable clustering. Figure 6A and B represents subject 2 left and right hemisphere respectively, whose modularity pattern did not reveal dramatic fragmentation. Subject 1 had a WAB-AQ score of 48.1 while subject 2 scored 88.1. Figures 7 and 8 show two example participants, subject 3 has percent white matter damage, lesion volume and location comparable to subject 2 (subject 3: 99.24 cm^3^, percent white matter damage: 0.096). Subject 3’s left hemisphere is however fragmented into 9 modules compared to subject 2’s 4 modules. This fragmentation occurs mainly in the inferior frontal temporal regions, with the same subject’s right hemisphere remaining relatively intact (4 modules – Fig. 7B). Subject 3 had a WAB-AQ score of 58.2 Subject 4 had behavioral and fragmentation patterns similar to subject 1 even with a significantly smaller lesion volume (subject 4: 206.36 cm^3^, percent white matter damage: 0.035). Subject 4 revealed a similar pattern of fragmentation in the left hemisphere, with a relatively intact right hemisphere (Fig. 8A and B respectively), and had a WAB score of 41.8.

**Figure 9:**
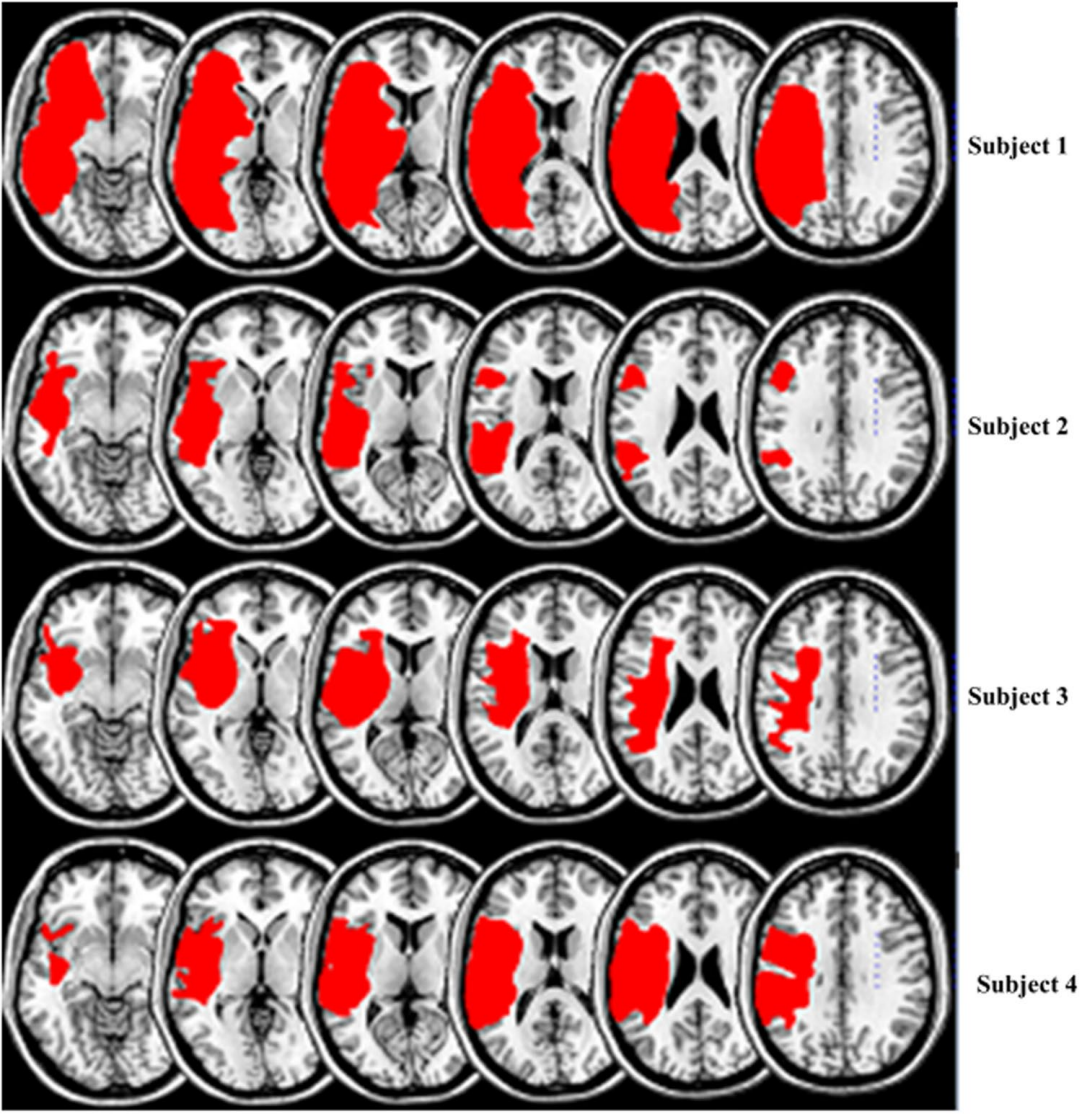
Overlap plot of damage locations in our 4 exemplar subjects. Subject 1: total lesion volume 359.4 cm^3^, Subject 2: total lesion volume 76.1 cm^3^, Subject 3: total lesion volume 99.24 cm^3^, Subject 4: total lesion volume 206.36 cm^3^.

Figure 9 shows the lesion locations of all 4 example subjects.

Supplementary Table 1 shows pairs of regions that when in the same module, are associated with a higher WAB-AQ score. Overall, they indicate that nodes in the temporal, inferior frontal, middle temporal and insular regions need to be more tightly associated in the context of the overall community structure for preservation of language.

### Relationship between Aphasia severity and cortical damage, modularity, and hubs

There was a significant relationship between WAB-AQ and total brain damage (F = 49.1, p < 0.00001, adjusted R^2^ = 0.351). When number of hubs was added as a predictor, the model composed of damage and number of hubs had equivalent explanatory power, and number of hubs was not a significant predictor of deficit (F = 25.7, p < 0.00001, adjusted R^2^ = 0.357; damage (p =  < 0.00001), hubs (p < 0.18)). With modularity added as a predictor, the model composed of damage and modularity had a higher explanatory power, with modularity being a significant predictor of deficit in addition to cortical damage (F = 27.4, p < 0.00001, adjusted R^2^ = 37.2; damage (p < 0.00001), modularity (p = 0.049)).

### Relationship between Aphasia severity and inter- and intra-module connectivity

**Figure 10:**
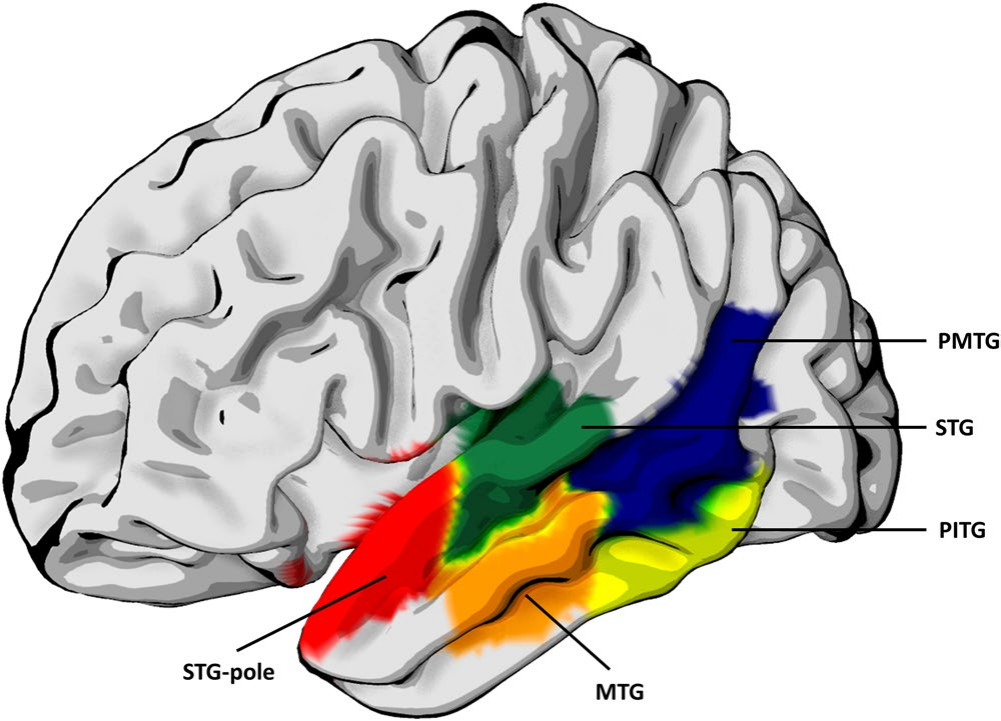
Regions from the language specific and domain general networks whose module sizes were significantly correlated with aphasia severity. STG-superior temporal gyrus (p = 0.0053), STG-pole -the pole of the superior temporal gyrus (0.0054), MTG- middle temporal gyrus (p = 0.0042), PMTG- posterior middle temporal gyrus (p = 0.0448), and PITG- posterior inferior temporal gyrus (p = 0.0123).

There was a significant relationship between aphasia severity and the reduced size of modules containing language specific regions in the temporal lobe. The superior temporal gyrus (p = 0.0053), the pole of the superior temporal gyrus (0.0054), the middle temporal gyrus (p = 0.0042), the posterior middle temporal gyrus (p = 0.0448), and the posterior inferior temporal gyrus (p = 0.0123) we all significantly correlated with WAB-AQ as shown on Fig. 10. (Not corrected for multiple comparison). Nodes from the domain general network did not significantly correlate with aphasia severity.

### Pars triangularis

There was a significant relationship between intra-modular degree and WAB-AQ (r = 0.265, p = 0.0058), and between the node’s participation coefficient and WAB-AQ (r = 0.4848, p < 10^-5^). Participation coefficient survived partial correlation with module specific damage (r = 0.271, p = 0.0051). Intra modular degree did not survive partial correlation. There was no significant correlation between module size and WAB-AQ.

### Pars opercularis

There was a significant relationship between intra-modular degree and WAB-AQ (r = 0. 3520, p < 0.0001), and between the node’s participation coefficient and WAB-AQ (r = 0.4308, p < 0.00001). Participation coefficient survived partial correlation with module specific damage (r = 0.2865, p = 0.0032). Intra modular degree did not survive partial correlation. Module size was significantly correlated with WAB-AQ (r = 0.2073, p = 0.0250).

### Superior temporal gyrus

There was a significant relationship between intra-modular degree and WAB-AQ (r = 0.5112, p < 10^−7^), and between the node’s participation coefficient and WAB-AQ (r = 0. 3915, p < 0.00001). Both participation coefficient (r = 0.2112, p = 0.0235) and intra modular degree (r = 0.2898, p = 0.0029) survived partial correlation with module specific damage.

Module size was significantly correlated with WAB-AQ (r = 0. 2538, p = 0.0079), and also survived partial correlation with module specific damage (r = 0.1782, p = 0.0474).

## Discussion

The primary purpose of the current study was to determine the degree to which post stroke fragmentation of brain anatomical connectivity affects language ability after stroke. To investigate this hypothesis, we analyzed the anatomical connectome from a large cohort of chronic stroke survivors with left hemisphere focal damage, and employed graph theory methods to assess the community structure of global and peri-Sylvian networks. We hypothesized that the fragmentation of the brain community structure and the disintegration of peri-Sylvian networks, even when these regions are relatively spared, would be associated with worse chronic aphasia. Our findings strongly supported our hypothesis: fragmentation to the brain neuronal network community structure, even when the cortical structures were relatively spared, were directly associated with more severe aphasia in the chronic period.

These results have direct implications for a better understanding of the mechanisms associated with post-stroke language recovery, as well as the relationship between neuronal network integrity and complex cognitive functions. Network modularity is one of the hallmarks of complex biological systems. It confers computational advantages, efficient processing, and robust responses to perturbations^24^. Modularity represents a fine balance between integration and segregation where both extremes can lead to poorly efficient networks. Very high modularity can lead to disintegrated or fragmented networks, while very low modularity can lead to lack of specialization. In our cohort, we observed that stroke lesions are associated with higher mean modularity in the lesioned hemisphere (μ = 0.4226) compared to the intact hemisphere (μ = 0.3450), suggesting that anatomical damage caused by the stroke is not only related to regional destruction, but may affect the entire organization of the remaining neuronal network architecture, resulting in a more segregated organization that hinders communication between modules. The clinical impact of this measured change in modularity was confirmed by the fact that subjects with very high modularity scores had more severe chronic aphasia, even when controlling for degree of cortical lesion.

Post-stroke high modularity scores are likely related to a combination of increased local clustering or modular fragmentation, weaker inter-modular integration, and, as our group previously demonstrated in post-stroke damage, loss of connectivity hubs^23^. These variables lead to less efficient information transfer.

It is important to acknowledge that we used each participant’s own right hemisphere as a control, which assumes anatomical integrity of the right hemisphere. This is a limitation for two reasons. First, is assumes that the right hemisphere is relatively preserved after the stroke, when in fact there could be remodeling due to deafferentation or demand for compensation due to loss of function. Second, it does not consider the physiological asymmetries in anatomical connectivity. It would therefore be essential to determine the reliability of the right hemisphere as a self-hemispheric control. Despite this potential limitation, there is an inherent advantage of using the subject’s own non-lesioned hemisphere anatomy as the control, since it considers the relative disruption of network topology when controlling for other issues such as age, pre-stroke microangiopathic white matter loss and global network organization, which affect both hemispheres equally. Furthermore the right hemisphere re-organization has been implicated in aphasia recovery^25^ and therefore assessing the degree of re-organization in relation to the lesioned hemisphere maybe more informative than comparison to healthy controls. For this reason, this approach was successful to demonstrate a strong and significant correlation between the left hemisphere community affiliation index and WAB-AQ, indicating that language was preserved if more regions were consistently grouped in the same module and not segregated across modularity optimization runs.

While modularity is a measure of global network organization, the mutual participation of key regions into the same module is of potential interest. Thus, we identified pairs of regions that when in the same module, were associated with a higher WAB-AQ score (supplementary Table 1). We did not explore the typical composition of modules beyond two pairs but were still able to observe consistent pairing of the classical language regions, as well as other cortical and insular regions in patients who did not have severe aphasia. The impact of changes of the typical composition of modules (with two or more regions) may be a topic for further focused study. Furthermore, we acknowledge that we did not perform direct out of sample prediction, perhaps a more informative measure of recovery, to determine the predictive power of modularity, we still propose this to be an important first step in the understanding of topological changes post-stroke.

Importantly, our results suggest that connectome community structure may be a very useful, personalized, and unique score to inform about language prognosis after stroke. Currently, our ability to predict aphasia recovery is still suboptimal and tied to lesion size and location even though evidence points to the reorganization of the remaining brain networks beyond the lesion as being crucial for recovery. Our results show a significant correlation between disintegration of the community structure and long-term aphasia severity, providing an indication into one of the many determinants of the biological substrates of stroke recovery. There are no other methods that can provide an accurate description of post-stroke prognosis regarding language. This may be the optimal usage of the connectome community structure, which can inform clinicians and patients about the magnitude of brain framework damage. Furthermore, this is an optimal approach for clinical translation of computational neuroscience because the connectome community structure provides a unique and yet abridge window to the basic framework from which complex cognitive functions arise, i.e., the systems organization of neuronal networks.

In conclusion, we confirm that preservation of anatomical white matter network architecture is directly related to long-term aphasia severity. Loss of white matter integrity, even when the cortical regions are preserved, is associated with more severe aphasia. Modularity provides a single index that indicates the integrity of system-level organization of neuronal networks.

## Electronic supplementary material

Supplementary information
