# Supplementary material for: Chronic post-stroke aphasia severity is determined by fragmentation of residual white matter networks
Source: Sci Rep. 2017 Aug 15;7:8188. doi: 10.1038/s41598-017-07607-9 (PMC5558035; doi:10.1038/s41598-017-07607-9)
Supplement: Supplementary file 1 — Supplementary information [file 41598_2017_7607_MOESM1_ESM.pdf]

Chronic post-stroke aphasia severity is determined by fragmentation of residual white matter networks.

Barbara K Marebwa[1], Julius Fridriksson[2], Grigori Yourganov[3], Lynda Feenaughty[1,2], Chris Rorden[3], Leonardo Bonilha[1].

Supplemental Material

Supplementary table S1 - Pairs of regions that when in the same module, are associated with a higher WAB-AQ score, Bonferroni corrected at p< 0.05. Overall, they indicate that temporal, inferior frontal, medial temporal and insular regions need to be connected for preservation of language.

ROI 1	-	ROI 2	p values
superior frontal gyrus (posterior segment)	-	inferior frontal gyrus pars opercularis	1.97E-05
middle frontal gyrus (posterior segment)	-	inferior frontal gyrus pars opercularis	7.77E-08
middle frontal gyrus (posterior segment)	-	inferior frontal gyrus pars triangularis	5.57E-06
inferior frontal gyrus pars opercularis	-	inferior frontal gyrus pars triangularis	1.95E-07
inferior frontal gyrus pars opercularis	-	postcentral gyrus	9.48E-08
inferior frontal gyrus pars opercularis	-	precentral gyrus	6.33E-08
inferior frontal gyrus pars opercularis	-	supramarginal gyrus	9.56E-06
inferior frontal gyrus pars triangularis	-	supramarginal gyrus	1.82E-05
inferior frontal gyrus pars opercularis	-	angular gyrus	5.10E-07
lateral fronto-orbital gyrus	-	superior temporal gyrus	7.52E-06
middle fronto-orbital gyrus	-	superior temporal gyrus	3.98E-09
gyrus rectus	-	superior temporal gyrus	3.16E-08
superior temporal gyrus	-	pole of superior temporal gyrus	1.25E-09
lateral fronto-orbital gyrus	-	middle temporal gyrus	1.11E-05
gyrus rectus	-	middle temporal gyrus	6.70E-08
superior temporal gyrus	-	middle temporal gyrus	9.29E-09
pole of superior temporal gyrus	-	middle temporal gyrus	2.67E-06
lateral fronto-orbital gyrus	-	pole of middle temporal gyrus	1.32E-06
gyrus rectus	-	pole of middle temporal gyrus	2.44E-08
superior temporal gyrus	-	pole of middle temporal gyrus	1.14E-08
pole of superior temporal gyrus	-	pole of middle temporal gyrus	2.30E-06

lateral fronto-orbital gyrus	-	inferior temporal gyrus	1.11E-05
gyrus rectus	-	inferior temporal gyrus	2.71E-08
superior temporal gyrus	-	inferior temporal gyrus	4.61E-09
pole of superior temporal gyrus	-	inferior temporal gyrus	8.96E-06
middle temporal gyrus	-	inferior temporal gyrus	2.03E-05
pole of middle temporal gyrus	-	inferior temporal gyrus	2.64E-05
gyrus rectus	-	parahippocampal gyrus	4.53E-07
superior temporal gyrus	-	parahippocampal gyrus	1.02E-06
lateral fronto-orbital gyrus	-	entorhinal are	1.63E-05
gyrus rectus	-	entorhinal area	6.14E-06
lateral fronto-orbital gyrus	-	fusiform gyrus	1.11E-05
gyrus rectus	-	fusiform gyrus	2.03E-07
superior temporal gyrus	-	fusiform gyrus	3.43E-09
gyrus rectus	-	middle occipital gyrus	2.06E-05
gyrus rectus	-	inferior occipital gyrus	1.29E-05
superior temporal gyrus	-	inferior occipital gyrus	1.58E-06
gyrus rectus	-	lingual gyrus	6.23E-07
superior temporal gyrus	-	lingual gyrus	3.30E-07
middle temporal gyrus	-	lingual gyrus	2.96E-05
superior temporal gyrus	-	subcallosal anterior cingulate gyrus	2.71E-06
middle temporal gyrus	-	subcallosal anterior cingulate gyrus	4.41E-06
pole of middle temporal gyrus	-	subcallosal anterior cingulate gyrus	9.93E-07
inferior temporal gyrus	-	subcallosal anterior cingulate gyrus	1.04E-06
fusiform gyrus	-	subcallosal anterior cingulate gyrus	1.04E-06
lingual gyrus	-	subcallosal anterior cingulate gyrus	1.47E-05
superior temporal gyrus	-	subgenual anterior cingulate gyrus	3.16E-08
middle temporal gyrus	-	subgenual anterior cingulate gyrus	6.70E-08
pole of middle temporal gyrus	-	subgenual anterior cingulate gyrus	2.44E-08
inferior temporal gyrus	-	subgenual anterior cingulate gyrus	2.71E-08
parahippocampal gyrus	-	subgenual anterior cingulate gyrus	4.53E-07
entorhinal area	-	subgenual anterior cingulate gyrus	6.14E-06
fusiform gyrus	-	subgenual anterior cingulate gyrus	2.03E-07
inferior occipital gyrus	-	subgenual anterior cingulate gyrus	1.29E-05
lingual gyrus	-	subgenual anterior cingulate gyrus	6.23E-07
middle frontal gyrus (dorsal prefrontal cortex)	-	insular	1.35E-05
lateral fronto-orbital gyrus	-	insular	2.63E-08
middle fronto-orbital gyrus	-	insular	6.65E-08
gyrus rectus	-	insular	3.49E-07
superior temporal gyrus	-	insular	6.95E-07
middle occipital gyrus	-	insular	1.10E-05
lingual gyrus	-	Insular	5.66E-06
subgenual anterior cingulate gyrus	-	Insular	5.69E-07

superior temporal gyrus	-	Amygdala	2.35E-07
gyrus rectus	-	hippocampus	8.56E-07
superior temporal gyrus	-	hippocampus	2.51E-08
subgenual anterior cingulate gyrus	-	hippocampus	8.56E-07
superior temporal gyrus	-	caudate nucleus	5.29E-08
middle temporal gyrus	-	caudate nucleus	9.89E-08
pole of middle temporal gyrus	-	caudate nucleus	3.67E-08
inferior temporal gyrus	-	caudate nucleus	4.00E-08
parahippocampal gyrus	-	caudate nucleus	5.86E-07
fusiform gyrus	-	caudate nucleus	2.62E-07
middle occipital gyrus	-	caudate nucleus	8.43E-06
inferior occipital gyrus	-	caudate nucleus	1.82E-06
lingual gyrus	-	caudate nucleus	8.06E-07
insular	-	caudate nucleus	5.16E-06
hippocampus	-	caudate nucleus	1.13E-06
pole of superior temporal gyrus	-	putamen	2.34E-06
parahippocampal gyrus	-	putamen	6.92E-08
entorhinal area	-	putamen	1.06E-05
amygdala	-	putamen	4.94E-06
hippocampus	-	putamen	2.84E-05
superior temporal gyrus	-	nucleus innominata of mynert	6.72E-09
insular	-	nucleus innominata of mynert	8.12E-08
superior temporal gyrus	-	nucleus accumbens	4.17E-12
insular	-	nucleus accumbens	6.76E-07
inferior frontal gyrus pars opercularis	-	cerebellum	3.69E-07
inferior frontal gyrus pars opercularis	-	medulla	1.83E-10
middle frontal gyrus (posterior segment)	-	posterior insula	5.25E-06
inferior frontal gyrus pars opercularis	-	posterior insula	2.60E-07
middle fronto-orbital gyrus	-	posterior insula	8.63E-13
angular gyrus	-	posterior insula	1.50E-09
pre-cuneus	-	posterior insula	6.92E-06
pole of superior temporal gyrus	-	posterior insula	3.58E-12
middle temporal gyrus	-	posterior insula	5.30E-10
pole of middle temporal gyrus	-	posterior insula	4.38E-11
inferior temporal gyrus	-	posterior insula	5.30E-10
parahippocampal gyrus	-	posterior insula	8.15E-13
entorhinal area	-	posterior insula	9.39E-12
fusiform gyrus	-	posterior insula	9.42E-08
middle occipital gyrus	-	posterior insula	1.71E-06
inferior occipital gyrus	-	posterior insula	2.53E-05
lingual gyrus	-	posterior insula	9.35E-07
amygdala	-	posterior insula	3.53E-13

Hippocampus	-	posterior insula	6.39E-09
nucleus innominata of mynert	-	posterior insula	2.26E-13
nucleus accumbens	-	posterior insula	3.33E-12
superior temporal gyrus	-	posterior middle temporal gyrus	7.21E-08
caudate nucleus	-	posterior middle temporal gyrus	2.01E-05
posterior insula	-	posterior middle temporal gyrus	2.20E-06
gyrus rectus	-	posterior inferior temporal gyrus	2.71E-08
superior temporal gyrus	-	posterior inferior temporal gyrus	8.66E-10
subcallosal anterior cingulate gyrus	-	posterior inferior temporal gyrus	1.04E-06
subgenual anterior cingulate gyrus	-	posterior inferior temporal gyrus	2.71E-08
caudate nucleus	-	posterior inferior temporal gyrus	4.00E-08
posterior insula	-	posterior inferior temporal gyrus	6.56E-09

Supplementary table S2 – Labels of brain regions included in the analysis

**index**	**-**	**Label**
1	-	superior frontal gyrus (posterior segment)
2	-	superior frontal gyrus (prefrontal cortex)
3	-	superior frontal gyrus (frontal pole)
4	-	middle frontal gyrus (posterior segment)
5	-	middle frontal gyrus (dorsal prefrontal cortex)
6	-	inferior frontal gyrus pars opercularis
7	-	inferior frontal gyrus pars orbitralis
8	-	inferior frontal gyrus pars triangularis
9	-	lateral fronto-orbital gyrus
10	-	middle fronto-orbital gyrus
11	-	gyrus rectus
12	-	postcentral gyrus
13	-	precentral gyrus
14	-	Superior parietal gyrus
15	-	supramarginal gyrus
16	-	angular gyrus
17	-	pre-cuneus
18	-	superior temporal gyrus
19	-	pole of superior temporal gyrus
20	-	middle temporal gyrus
21	-	pole of middle temporal gyrus
22	-	inferior temporal gyrus
23	-	parahippocampal gyrus
24	-	entorhinal area
25	-	fusiform gyrus
26	-	superior occipital gyrus

27	-	middle occipital gyrus
28	-	inferior occipital gyrus
29	-	cuneus
30	-	lingual gyrus
31	-	rostral anterior cingulate gyrus
32	-	subcallosal anterior cingulate gyrus
33	-	subgenual anterior cingulate gyrus
34	-	dorsal anterior cingulate gyrus
35	-	posterior cingulate gyrus
36	-	insular
37	-	amygdala
38	-	hippocampus
39	-	caudate nucleus
40	-	putamen
41	-	globus pallidus
42	-	thalamus
43	-	hypothalamus
44	-	nucleus innominata of mynert
45	-	nucleus accumbens
46	-	red nucleus
47	-	substancia nigra
48	-	cerebellum
49	-	cerebral peduncle
50	-	midbrain
51	-	pons
52	-	medulla
53	-	mammillary body
54	-	posterior insula
55	-	posterior superior temporal gyrus
56	-	posterior middle temporal gyrus
57	-	posterior inferior temporal gyrus

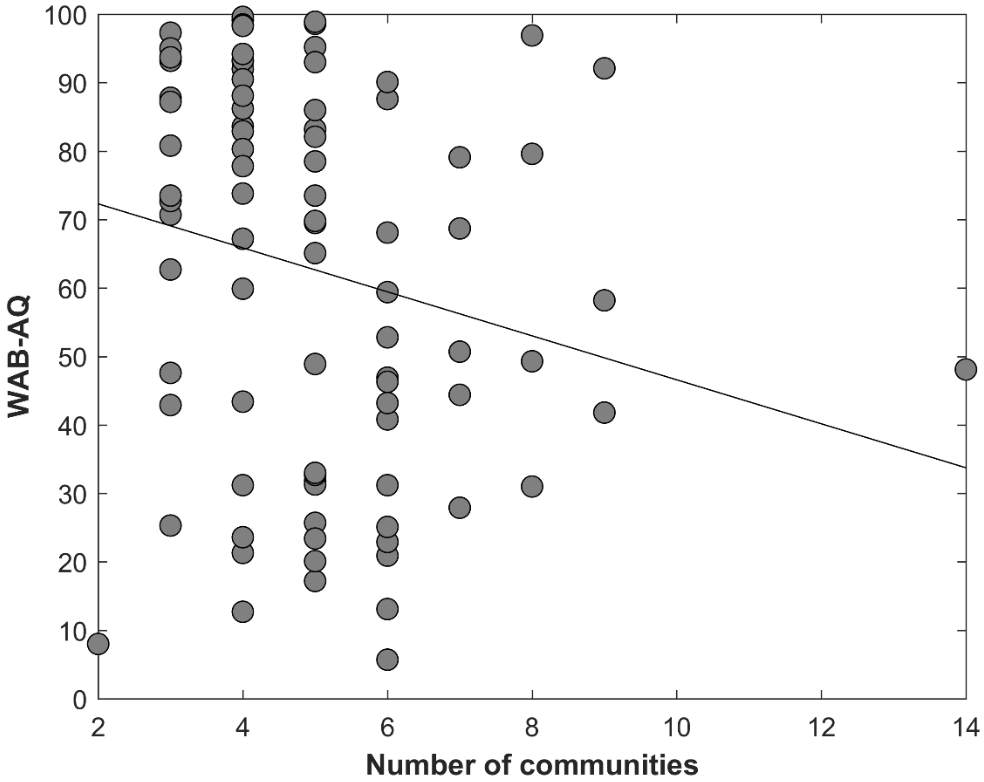

Supplementary figure 1. Correlation between number of communities in the left hemisphere and WAB-AQ ($r = -0.2036$, $p = 0.0271$). These correlations did not survive partial correlations accounting for grey or white matter damage.

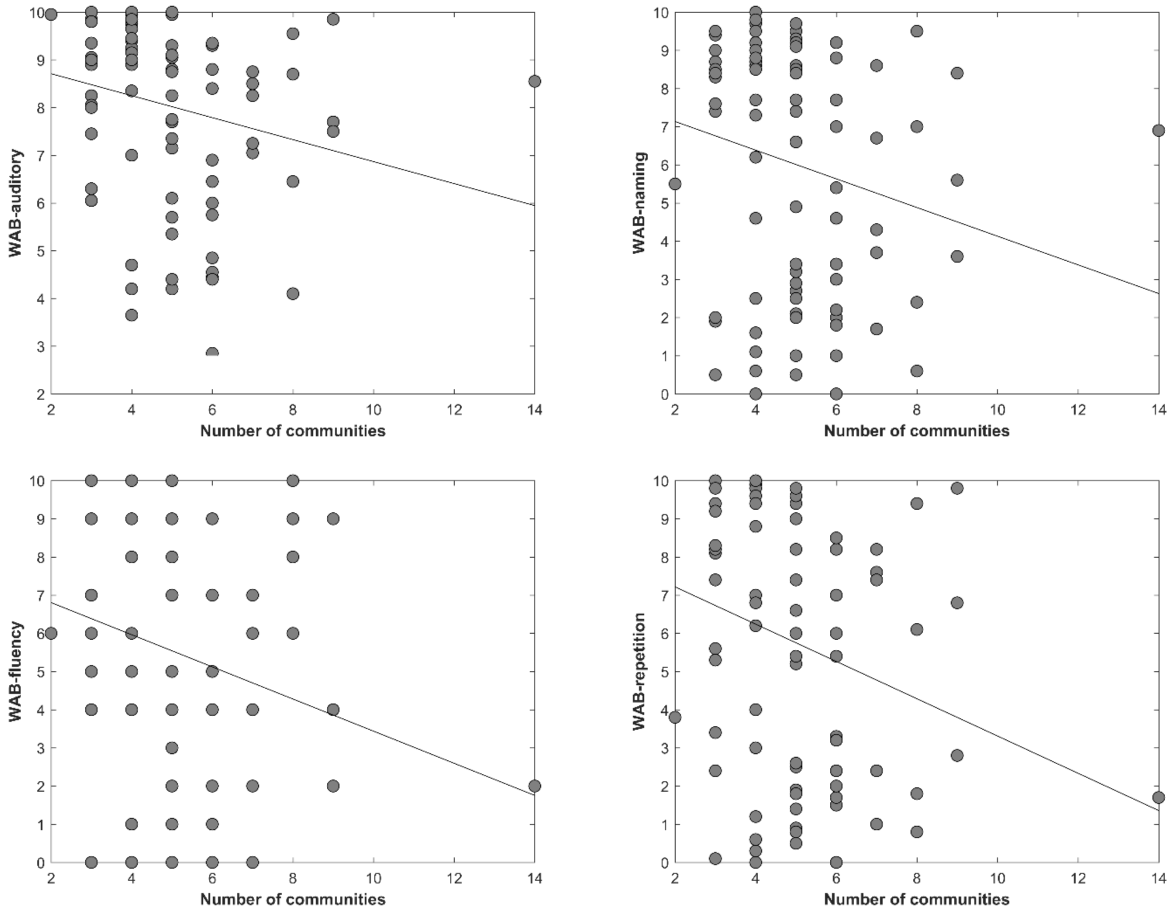

Supplementary figure 2. Correlation between number of communities in the left hemisphere and subsets of WAB-AQ: Auditory comprehension (r = -0.2149, p = 0.0210), Fluency (r = -0.2244, p = 0.0167), Naming (r = -0.2048, p = 0.0264), and Repetition (r = -0.2531, p = 0.0081). These correlations did not survive partial correlations accounting for grey or white matter damage.

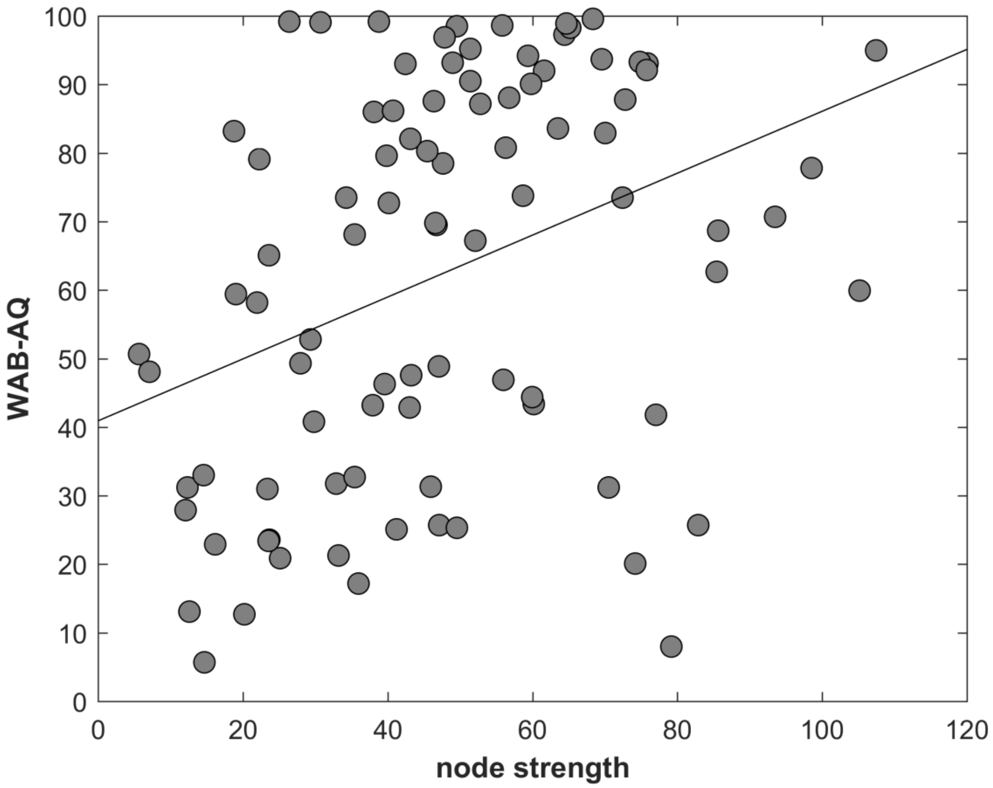

Supplementary figure 3. Correlation between lefth hemisphere node strength and WAB-AQ ($r = 0.3625$, $p < 0.0001$). These correlations did not survive partial correlations accounting for grey or white matter damage.

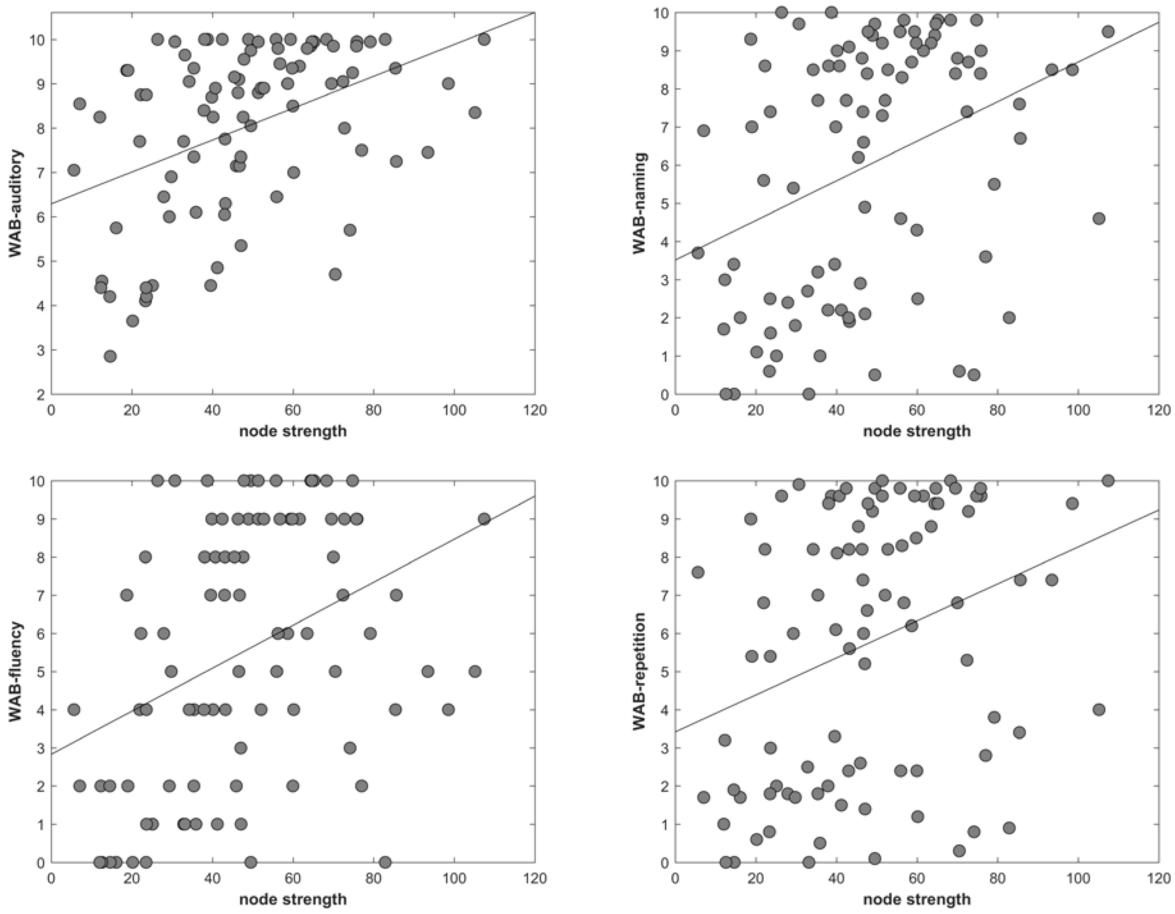

Supplementary figure 4. Correlation between left hemisphere node strength and subsets of WAB-AQ: Auditory comprehension ($r = 0.4251$, $p < 0.0001$), Fluency ($r = 0.3803$, $p < 0.0001$), Naming ($r = 0.3585$, $p < 0.0001$), and Repetition ($r = 0.3182$, $p = 0.0011$). These correlations did not survive partial correlations accounting for grey or white matter damage.
